# Supplementary material for: Pregnant women’s intentions to use maternity waiting homes and its associated factors in rural districts of Hadiya Zone, Southern Ethiopia
Source: PLoS One. 2023 Jun 2;18(6):e0281652. doi: 10.1371/journal.pone.0281652 (PMC10237373; doi:10.1371/journal.pone.0281652)
Supplement: S1 File — (DOCX) [file pone.0281652.s001.docx]

# Annex 1. Participant’s consent formant

Good morning/afternoon;

Hello. My name is -----------------------------I am interviewing participants in this zone to assess the reasons for pregnant women's intentions to use a maternal waiting home in rural Hadiya district, Southern Ethiopia.

Dear respondents;

Thank you for being cooperative to answer for the valid and effective completion. All what you have told will be kept in secret and you are not expected to tell your name and your name is not going to be registered. A code number will identify every participant. The interview is voluntary; you have the right to participate or not to participate at any time during the interview. Your refusal will not have any effect on the services that you or any of your family receives. Your participation is important to improve Maternal and neonatal health in your community as well as a country as a whole by increasing institutional delivery by implementing different intervention on MWH utilization .Therefore; you are kindly requested to give genuine answers according to the questions. You can ask questions to the interviewer for clarification.

Risk and /or Discomfort

If you are participating in this research, there may not be direct benefit to you but your participation is likely to help us in showing the proportion of women utilize MWH that help in decision making for stakeholders

Right to Refusal

You have full right to refuse from participating in this research and this will not affect you from getting any kind of health service.

This research project will be reviewed and approved by the ethical committee of the Hosana college of Health Science If you want to know more information you can contact through the address below. If you have any question you can contact any of the following individuals and you may ask at any time you want.

1. Mr .Habtamu Hasen MSc lecturer at Hosanna College of Health Science

Mobile: +25138641297 email: habtamu130@gmail.com

Is the information/ objective clear and willing to participate in the interview?

1) Yes 2) No

Thank you!

# **Annex II: English version Questionnaire**

Instruction: This is a structured questionnaire designed for the study of maternal waiting home utilization among women living in rural Hadiya zone, southern, Ethiopia, 2020.

*Note: Get consent before interviewing performing any activity.*

Questionnaire ID No/code _______

Date of interview____________________

Woreda_________________Kebele________________Got__________Village______________

| Part A: Socio-demographic information | | | | |
| --- | --- | --- | --- | --- |
| Q. No | Question | Codes | | Go to q. |
| A1 | Have you give birth in the last 6 month? | 1.Yes  0. No | | If no stop here |
| A2 | How old are you(completed  in Year)? | ------------years | |  |
| A3 | Where is your place of residence? | 1.Rural  2.Semi-urban/rural town | |  |
| A4 | What is your level of education? | 1-Illitrate  2-can read and write  3-Grade1-8  4-secondary and above | |  |
| A5 | What is your occupation? | 1-House wife  2-Farmer  3-Marchant  4-House maid  5-Government employee  6-Others specify------------ | |  |
| A6 | What is your religion? | 1. Orthodox  2. Muslim  3. Protestant  4. catholic  5. Other specify_____) | |  |
| A7 | What is your current marital status? | 1-Married  2-Never married  3-Seprate  4-Divorced  5-Widowed | |  |
| A8 | What is your ethnicity? | 1. Hadiya 2. Kembata 3. Silite 4. Guraghe 5. Others specify------------------- | |  |
| A9 | What was your age at first  marriage | ----------------years | |  |
| A10 | What is the level of education of your husband (for those married  only) ? | 1-Illitrate  2-Can read and write  3-1-8 grade  4-Secondary and above | |  |
| A11 | Occupation of your husband(for  those married only) | 1-Farmer  2-Marchant  3-Government employee  4-Daily laborer  5-other specify------------ | |  |
| A12 | How many of you are living in your house hold | -------------number | |  |
| A13 | Who is the decision maker in the house hold | 1-Self  2-Husband  3-Self and husband  4-Others,specify------------- | |  |
| A14 | What time it take from your home to health center /hospital in minute: | -----------------------minutes | |  |
| A15 | Prior Place of delivery | 1. Home 2. Health institution | |  |
| A16 | Have you ever had a child less than 5 years | 1.Yes  0. No | |  |
| Part B: Obstetric and antenatal experience of women | | | | |
| B1 | Did you attend ANC during your recent pregnancy | 1.Yes  2. No | |  |
| B2 | Age at first pregnancy |  | |  |
| B3 | Did you attend ANC ? | 1.Yes  0. No | | 0=B7 |
| B4 | At what month did you start  You’r ANC? | --------------- month | |  |
| B5 | How many times you attend ANC in your recent pregnancy? | ------------------times | |  |
| B6 | Where did you have ANC? | 1.health post  2. health center  3. hospital  4. other specify---- | |  |
| B7 | Do you ever had abortion or  miscarriage | 1.Yes  0. No | |  |
| B8 | Do you ever had still birth | 1.Yes  0. No | |  |
| B9 | How many Gravidity | --------------number | |  |
| B10 | How many Parity | __________number | |  |
| B11 | Did you have previous C/S History | 1.Yes  0. No | |  |
|  |  |  | |  |
| PART D: Health facility-related factors | | | | |
| D1 | Have you ever heard of a Maternity Waiting Home? | | 1.Yes  0. No |  |
| D2 | Was the mother admitted to MWH? | | 1.Yes  0. No | 0=D6 |
| D3 | How long had she been in the MWH? (Fill in completed days. | | --------------------days |  |
| D4 | Past experience of MWH | | 1.Yes  0. No |  |
| D5 | What do you think the advantages are of staying at a MWH? | | 1. Closeness to emergency obstetrics care 2. Saving life of mother 3. Saving life of baby 4. Calmness, rest before delivery 5. Other, specify |  |
| D6 | If answer for D4 is No What is your reason for not going to the hospital (for MCH services)? | | 1. Cost too much 2. Facility not open 3. Too far/no transportation 4. Don’t trust facility 5. Poor quality of service 6. No female provider at facility 7. Husband/family did not allow 8. Not necessary 9. Not customary 10. Other, specify |  |

Part G: Directions: The following items examine some of intention to use maternal waiting home among pregnant women. Please circle the number, which most accurately represents your feelings about these intentions.

| Q.  No |  | Very unlikely | unlikely | Not sure | Likely | Very likely |
| --- | --- | --- | --- | --- | --- | --- |
| G1 | I am intended to stay in health center for institutional delivery 15 days before giving birth: | 1 | 2 | 3 | 4 | 5 |
| G2 | I will try to stay in health center for institutional delivery 15 days before giving birth | 1 | 2 | 3 | 4 | 5 |
| G3 | want to stay in health center for institutional delivery 15 days before giving birth | 1 | 2 | 3 | 4 | 5 |
| G4 | I like to stay in health center for institutional delivery 15 days before giving birth |  |  |  |  |  |

Indirect measurement attitude

Directions: The following items examine some of belief about donate blood on the next six month in blood bank. Please circle the number, which most accurately represents your feelings about this donating belief.

| No | Behavioral belief | Strongly disagree | disagree | Not sure | agree | Strongly agree |
| --- | --- | --- | --- | --- | --- | --- |
| I1 | Staying in health center for institutional delivery 15 days before giving birth will help me to  get delivery by health professionals & prevent myself from death related to delivery | 1 | 2 | 3 | 4 | 5 |
| I2 | Staying in health center for institutional delivery 15 days before giving birth will help me to  get healthy child: | 1 | 2 | 3 | 4 | 5 |
| I3 | Staying in health center for institutional delivery 15 days before giving birth will help be to  be happy & reduce fear of labor | 1 | 2 | 3 | 4 | 5 |
|  | Staying in health center for institutional delivery 15 days before giving birth will help me to  get better ANC services: | 1 | 2 | 3 | 4 | 5 |
|  |  |  |  |  |  |  |
| I4 | Staying in health center for institutional delivery 15 days before giving birth will help me to  get better health information on child immunization, family planning & personal hygiene | 1 | 2 | 3 | 4 | 5 |
|  |  |  |  |  |  |  |

|  | Outcome evaluation | Strongly disagree | disagree | Not sure | agree | Strongly agree |
| --- | --- | --- | --- | --- | --- | --- |
| O1 | For me getting delivery by health professionals & prevent myself from death related to delivery | 1 | 2 | 3 | 4 | 5 |
| O2 | For me getting healthy child is good | 1 | 2 | 2 | 4 | 5 |
| O3 | For me being happy & reduce fear of labor is good | 1 | 2 | 2 | 4 | 5 |
| O4 | For me getting better ANC services is good | 1 | 2 | 2 | 4 | 5 |
| O5 | For me getting health information on immunization, family planning & other health service  Is important |  |  |  |  |  |

Part 4: Directions: How would the following people or groups can influence your intention to use maternal waiting home? Please circle the number that most actually represents your feelings about these statements.

| Q  No | Normative belief | Strongly disagree | Disagree | Not sure | agree | Strongly agree |
| --- | --- | --- | --- | --- | --- | --- |
| J1 | My mother thinks that I should stay in health center for institutional delivery 15 days before giving birth: | 1 | 2 | 3 | 4 | 5 |
| J2 | My husband thinks that I should stay in health center for institutional delivery 15 days  before giving birth | 1 | 2 | 3 | 4 | 5 |
| J3 | My religion leaders thinks that I should stay in health center for institutional delivery 15 days  before giving birth | 1 | 2 | 3 | 4 | 5 |
| J4 | My neighbor thinks that I should stay in health center for institutional delivery 15 days  before giving birth | 1 | 2 | 3 | 4 | 5 |
|  |  |  |  |  |  |  |
| J5 | Health extension workers approve my staying in health center for institutional delivery 15  days before giving birth |  |  |  |  |  |
|  | Motivation to comply | Strongly disagree | disagree | Not sure | agree | Strongly agree |
| M1 | My mother’s approval of staying in health center for institutional delivery 15 days before  giving birth is important to me | 1 | 2 | 3 | 4 | 5 |
| M2 | My husband’s approval of staying in health center for institutional delivery 15 days before  giving birth is important to me | 1 | 2 | 3 | 4 | 5 |
| M3 | My religion leaders approval of staying in health center for institutional delivery 15 days before  giving birth is important to me | 1 | 2 | 3 | 4 | 5 |
| M4 | My neighbors’ approval of staying in health center for institutional delivery 15 days before  giving birth is important to me | 1 | 2 | 3 | 4 | 5 |
| M5 | Health extension worker’s approval of staying in health center for institutional delivery 15  days before giving birth is important to me | 1 | 2 | 3 | 4 | 5 |

Part 5: Please indicate your level of agreement with the following statements by circling the number that most closely matches your positions.

Indirect measure perceived behavioral control

| Q no |  | | Strongly disagree | | disagree | | Not sure | | agree | | Strongly  agree | |
| --- | --- | --- | --- | --- | --- | --- | --- | --- | --- | --- | --- | --- |
| P1 | When my gestational age advances, I cannot get transportation/not walk long distance/ to  go & stay in health center for institutional delivery prior to 15 days before giving birth. | | 1 | | 2 | | 3 | | 4 | | 5 | |
| P2 | If I stay in health center I could not get enough food to stay in health center for institutional  delivery 15 days before giving birth | | 1 | | 2 | | 3 | | 4 | | 5 | |
| P3 | If my gestation increases I cannot get individuals to take to health center for staying in it for  institutional delivery 15 days before giving birth | | 1 | | 2 | | 3 | | 4 | | 5 | |
| P4 | Difficult to get individuals that can give care for my family once I left to maternity waiting  home | | 1 | | 2 | | 3 | | 4 | | 5 | |
|  | | Power of control | | Strongly disagree | | disagree | | Not sure | | agree | | Strongly  agree |
| PC1 | | Lack of transportation /long distance/ makes it difficult for me to go & stay in health center  for institutional delivery prior to 15 days before giving birth: | | 1 | | 2 | | 3 | | 4 | | 5 |
| PC2 | | Food insecurity in health center makes it difficult for me to stay in health center for  institutional delivery 15 days before giving birth | | 1 | | 2 | | 3 | | 4 | | 5 |
| PC3 | | Hard ship of staying/desolate surrounding makes it more difficult for me to stay in health  center for institutional delivery 15 days before giving birth | | 1 | | 2 | | 3 | | 4 | | 5 |
| PC4 | | Difficulty to get individuals that can give care for my family makes it difficult for me to  stay in health center for institutional delivery prior to 15 days before giving birth | |  | |  | |  | |  | |  |

| SECTION W: Household Wealth  *Now I will ask you about some fixed assets that your household have* | | | |
| --- | --- | --- | --- |
| Does the household have any of the following properties? (Circle) | | Yes | No |
| W1 | Functioning radio/Tape recorder/CD player | 1 | 0 |
| W2 | Functioning Television | 1 | 0 |
| W3 | Gas Stove | 1 | 0 |
| W4 | Kerosene stove | 1 | 0 |
| W5 | Electric stove | 1 | 0 |
| W6 | Bicycle | 1 | 0 |
| W7 | Motor Cycle | 1 | 0 |
| W8 | Cart/Gari | 1 | 0 |
| W9 | Watch (Hand/Wall) | 1 | 0 |
| W10 | Mobile phone | 1 | 0 |
| W11 | Plough | 1 | 0 |
| W13 | Sofa | 1 | 0 |
