# Supplementary material for: Pregnant women’s intentions to use maternity waiting homes and its associated factors in rural districts of Hadiya Zone, Southern Ethiopia
Source: PLoS One. 2023 Jun 2;18(6):e0281652. doi: 10.1371/journal.pone.0281652 (PMC10237373; doi:10.1371/journal.pone.0281652)
Supplement: Attachment — Submitted filename: Manuscript Review_PONE-D-21-13771.doc [file pone.0281652.s003.doc]

**Manuscript Review # PONE-D-21-13771**

Thank you for the opportunity to review this interesting manuscript entitled “*Facilitators and barriers of pregnant women’s intention to use maternal waiting home in Southern Nations, Nationalities and Peoples Region, Ethiopia*”. The study presented in this manuscript is on an important and timely topic. It presents relevant information for the audience of this journal and would be of interest to its readership. I will first make some general comments and then add specific areas under each section recommended for revision.

**General Comments:**

This manuscript presents interesting and important information related to the maternity waiting home use in a rural part of Ethiopia. Prior to publication, the manuscript will need clarity in the methods and results sections. The authors could use the assistance of an editor for basic grammar and sentence structure corrections as well.

**Title:** The title is somewhat misleading. The authors report on factors that influence women’s intent to use a MWH prior to delivery. Barriers to use were not examined in what I can glean from the questionnaire nor from the reported results.

**Abstract:** The abstract is adequate. There are a few grammatical errors.

**Introduction:** Several sections of the introduction require attention. In the second paragraph, the author’s state: “the use of MWHs is part of a larger initiative to change the health system by providing a low cost alternative”. (lines 64-66). Low cost to what? The third paragraph, beginning on line 71-80 begins with a statement that MWHs are associated with a variety of risk factors that vary between developed and developing countries, although there is no support from the literature on which developing countries the authors are using for comparison. Lines 82-83 stat only a few studies have been conducted on women’s intention to use MWHs in the region. A quick search of the literature found four studies from Ethiopia that specifically examined women’s intention to use a MWH. Minimally, these should be cited in the introduction with a brief description of the findings.

**Materials and Methods:** The first sentence (line 95) describes the study as a community based cross-sectional study. It is not until later in the section that it is identified as a household survey. Please move this description closer to the beginning of the section. A clearer description of inclusion and exclusion criteria is needed. The author’s state: “participants who were seriously ill during data collection were excluded”. (lines 105-106). Please provide what was meant by “seriously ill”.

Lines 126-128 “The most common source of knowledge about MWHs was used to consider the source of information”. Please explain/clarify.

**Measurements:** It is not clear to the reader how the authors decided on a score of 60% or higher as a “favorable attitude toward MWHs”. Please provide more context. The same is needed for the “subjective norm” of 60% or higher – which I believe is from the section of questions in the survey that relate to others influence on the woman’s decision to use the MWH. This is extremely interesting information and an understanding by the readership would greatly add to the contribution this manuscript makes to our understanding of how women make decisions on whether or not to use a MWH.

**Data Collection Tools and Procedures:** Additional detail is needed on the procedure used to establish content validity of the tool. Lines 151-152 – “The questionnaires validity and reliability were determined in a scientific manner.” This sentence is not needed once the authors describe the process for the reader.

It is not clear to me if the participants filled out the questionnaire themselves or if the questions were read to the participants by the data collection team. Additional details on the data collection procedures are needed including the informed consent process. It is also not clear how the wealth index was calculated.

**Data Processing and Analysis:** Additional details on the data analysis are needed including how the authors chose the inclusion variables for the regression analyses and the dependent and independent variables for the odds ratio.

**Results:** With additional analysis and explanation of variables this section can be expanded to give a more detailed picture of the results.

**Discussion:** The authors set their findings in the context of other studies done within Ethiopia on intent to use a MWH adding to the impact of their findings. A more detailed limitations section is needed.

**Conclusion:** Adequate.

**References:** Adequate.
