# Supplementary material for: Pregnant women’s intentions to use maternity waiting homes and its associated factors in rural districts of Hadiya Zone, Southern Ethiopia
Source: PLoS One. 2023 Jun 2;18(6):e0281652. doi: 10.1371/journal.pone.0281652 (PMC10237373; doi:10.1371/journal.pone.0281652)
Supplement: Attachment — Submitted filename: Response to reviewers.docx [file pone.0281652.s004.docx]

**Thank you very much for your detail questions, comments and suggestions**

**Response to the editor**

1. Please ensure that your manuscript meets PLOS ONE's style requirements, including those for file naming.

**Response to editor:** We thankful for the given comment. Now we have tried to follow the PLoS one submission guideline format.

2. In your Data Availability statement, you have not specified where the minimal data set underlying the results described in your manuscript can be found.

**Response to editor:** Thank you for the comment. Now we have clearly described about the Data Availability statement. The editor may this part.

3. Please amend the manuscript submission data (via Edit Submission) to include author Manayeh Mulusew.

**Response to editor:** Thank you for the comment. Now we have corrected it according to the given comment.

**Additional Editor Comments**

1) Page 12, lines 100-102, providing some context/definitions on what the different administrative regions mean (e.g, kebeles, health posts)

**Response to editor:** Thank you for the comment. We share the raised concern. For more clarification we have added the definition of kebeles and health post. The reviewer may see the measurement part.

2) Page 13, lines 142-143 & Page 14, lines 152-156, more details are needed on the training for data collectors. How were they trained and what does it mean that they had close supervision? Did the data collectors complete the survey or did participants complete the survey or did it depend on literacy?

**Response to editor:** Thank you for the comment. Now we have made a great modification by adding statements on the result and tool part. The reviewer may see the result and the data collection tool part.

3) I also concur with Reviewer #1, that submitting the questionnaire is very valuable, but not all readers will review it. It is therefore important to provide examples of the survey items within the text.

**Response to editor:** Thank you for the comment.

1) Title: The title of the article is inaccurate. This article is not about barriers and facilitators to pregnant women’s intention to use MWHs. It is about the factors associated with that intention – Women’s individual-level characteristics and their obstetric history. Other articles have been published about the barriers and facilitators to use MWHs and usually have asked women qualitatively what their experience has been.

**Response to reviewer;** We are grateful for the given comment and we have made the essential modification on the title. The reviewer may see the title part.

2) Premise of article: All of the questions in the survey ask specifically about intention to use MWHs for 15 days but this is not brought up in the methods, discussion, or limitations. This aspect needs to be addressed. It could be considered a major limitation of the study as women who may have intended to use the MWH for only 10 days or even for 18 days, may have answered all of the intention to use questions in the negative because of how specific they were. While 15 days may be recommended by the Ethiopian Ministry of Health (assumption), it does not mean all women will follow this directive to the letter. I suggest including the 15 day aspect in the article title, explaining it in the methods, including it in table titles and subheadings, and discussing the implications of the 15 days in the discussion, including as a limitation. Include an explanation on why the 15 days was specifically chosen, how it relates to government policy or other MWH use guidelines if at all, and how it relates to the average length of stay of MWH users in Ethiopia and elsewhere.

**Response to reviewers;** Thank you for your deep insight. We understand the raised concern. But, to answer the question we have used the WHO statement about Maternity homes were set up in the vicinity of hospitals so that women from areas that were remote and/or difficult to access could be accommodated during the last two weeks or average of 15 days of pregnancy.
3) Introduction: Paragraph 2 of the introduction (or a small additional paragraph) should include findings to date on the impact of MWHs on maternal and neonatal health outcomes. While an important study in Ethiopia was cited, there are other articles which address the impact of MWH in sub-Saharan Africa on maternal mortality, facility delivery rates, stillbirths, and caesarean section rates. Lines 86-87 should be moved to this paragraph and include those citations.

**Response to reviewer 1:** Thank you for the comment. Correction is made according to the raised concern. The reviewer may see the introduction part.

4) Introduction: The last sentence of the introduction is passive. I suggest making it active and engaging. For example: “…sustainable development by providing relevant MWH information and advice to policy makers and implementers in Ethiopia.” See my point below on weaving this into the discussion.

**Response to reviewer 1:** Thank you for the comment. Correction is made according to the provided comment. Now we make it active. The reviewer may see the introduction part.

5) Methods: When discussing sampling, clarify and simplify the description. The sentences jump back and forth between source and sample populations. Describe the linear process followed from identifying the source population to selecting the sample

**Response to reviewer 1:** Thank you for the comment. Now we have made a considerable amendment. The reviewer may see the method part.

6) Methods: How were the education categories in the questionnaire chosen? These do not appear to be standard, such as what is used in the Demographic and Health Surveys. The authors should include a citation if they have one.

**Response to reviewer:** Thank you for the comment. Now we have corrected it according to standard classification of Demographic and Health Surveys. The reviewer may see table 1.
7) Methods: Give examples of questions included in the survey for intention to use MWHs, attitudes toward MWHs, and subjective norms toward MWHs. Not all readers will examine the supplemental questionnaire, so having an example of each in the text would be helpful. However, I appreciate the authors including the questionnaire, it was helpful to have.

**Response to reviewer:** Thank you for the comment. Now we have included the necessary elements.
8) Methods: Was gestation age considered in the exclusion criteria or in the analysis? It was asked in the survey but not included in the tables. What if the woman had not considered use of the MWH because they were only a few weeks pregnant? Gestational age of the respondents should be included in Table 1 and in the linear regression model if significantly associated with MWH intention.

**Response to reviewer:** Thank you for the comment. We understand the raised concern. We have made important corrections.

9) Methods: In the questionnaire, all behavioural belief and outcome evaluation questions were written in the positive and many sound leading. There are no negative views of the MWHs for participants to agree or disagree with. Include an explanation in the methods for how these questions were developed and why the authors chose to write them all in the positive. The authors should include discussion around the possibility of social desirability bias and acquiescence bias in the limitations section. Were the midwives who collected the data from within the health facilities accessible to the population? This could also increase social desirability bias if the participants knew them.

**Response to reviewer:** Thank you for the comment. Now the data collection tool is substantially modified to increase its clarity. The reviewer may see the method part.
10) Methods: The article would benefit from having a study setting to provide more information on the health system in this region of Ethiopia and the state of MWHs. Since the government is encouraging MWH use, has it also built new MWH structures or are the structures community constructed? Are MWHs only located at primary health centers, only at hospitals, or both?

**Response to reviewer:** Thank you for the comment. We understand the raised concern. In the tool part we want to say health facility not health center. However, MWHs are located both in health centers and hospitals. Generally, MWHs have been set up near hospitals with no facilities for operative deliveries, or near district or teaching hospitals with operative facilities. The set up chosen will depend on the

11) Discussion: In the first sentence of the discussion (lines 262-263), the authors write that the purpose of the research was to find out what factors influenced pregnant women’s decisions to use MWHs. This is not supported by the results. The authors only know what demographic characteristics about the women and their previous obstetric history are associated with intention to use MWHs. They do not know what goes into women’s decision-making regarding barriers they face to access, desire for a safe delivery, have permission or support from their partner, etc.

**Response to reviewer:** Thank you for the comment. We are indebted for the comment and we have removed unnecessary statements. The reviewer may see the introduction part of the discussion.
12) Discussion: The discussion is missing policy or implementer implications. There is a sentence about this in the conclusion that should be included in the discussion and expanded upon. What this research means in terms of suggestions for what Ethiopia’s national strategy regarding MWHs is a critical missing piece. Thank you for the comment. Based on the given comment the essential correction is made. The reviewer may see the discussion part.

**Response to reviewer:** Thank you for the comment.

13) The article requires English language editing and revisions specifically for sentence structure and grammar, particularly verb tense. The writing could generally be tightened up and shortened. The sampling and “factors associated with intentions to use MWHs” are two particular examples of sections that could be tightened up.

**Response to reviewer:** We are grateful the suggestion. Now we have used professional editing service like true editors and proofreading to correct spelling, punctuation and grammatical errors. Moreover, now we tried make it short and precise.
14) It would be helpful to provide the location of a cvs file for the data in addition to or instead of the SPSS file as not everyone uses or has access to SPSS. A csv file would allow even users with free software (such as R) to analyze the data. This does not need to be included as a supplementary file, though it can be. It could be housed in a data repository.

**Response to reviewer:** We are grateful for the suggestion. We will try to use data repository.

Minor revisions

1) General: Consistently use maternity waiting homes throughout the article and the title. The authors switch between maternal and maternity throughout the text when discussing the waiting homes.

**Response to reviewer:** Thank you for the comment. Now we have made the necessary amendment.
2) Abstract, Conclusions: “Almost half” should say “More than half”

**Response to reviewer:** Thank you for the comment. Now it is corrected as per the provided comment. The reviewer may see the abstract part.

3) Key words: Use key words not already included in the title of the paper to reach more readers. I suggest: obstetric care, maternal health, maternal waiting home, sub-Saharan Africa

**Response to reviewer:** Thank you for the comment. We accept the suggestion and we have an amendment. The reviewer may see the abstract part.

4) Introduction: What does the sentence beginning paragraph 3 mean? While the list of factors associated with MWH use is technically accurate, it is not engaging for the reader. Consider breaking it down into smaller sentences with logical groupings. For example, the authors could group by individual, interpersonal, and health systems factors.

**Response to reviewer:** Thank you for the comment. We understand the comment and we have modified it accordingly.

5) Methods: In the text and tables, clarify which pregnancy the questionnaire asked about. For example, for “Number of ANC visits”, it is unclear whether this is about the current pregnancy or the last one.

**Response to reviewer:** Thank you for the comment. Now we have corrected it. The reviewer may see table 2.

6) Methods: How were the reference categories chosen in Table 3.

**Response to reviewer:** Thank you for the comment. The reference was selected based on the nature of variables.

7) Methods: Lines 124 through 126 on principal component analysis should be moved to the analysis section and include a citation.

**Response to reviewer:** Thank you for the comment. We have made the necessary changes.

8) Methods: I am unclear what the sentence on lines 126 to 127 means. Please clarify.

**Response to reviewer:** Thank you for the comment. We have made a great modification in the method part as a whole. The reviewer may see the method part.

9) Methods: Lines 151 to 152: “The questionnaire’s validity and reliability was determined in a scientific manner.” The meaning of this sentence is unclear.

**Response to reviewer:** Thank you for the comment. No we have made substantial change regarding to the questionnaire. Additionally, we have removed vague statements. The reviewer may see the method part

10) Table 1: How was time to reach health facility determined and by what transport method? Were women asked times without reference to transport method? Clarify in the methods and in the indicator title in Table 1.

**Response to reviewer:** We are thankful for the comment. Now we have made the essential change. The reviewer may see Table 1.

11) Table 1: What does “Number of households” mean? Does this mean number of household members? Clarify.

**Response to reviewer:** Thank you for the comment. Now we have made the necessary change the reviewer may see table 1.

12) Results: Include the table reference in parentheses, in the first sentence in which data from the table is described. For example, the reference to Table 3 should be in the first sentence of the sub-section, not the last.

**Response to reviewer:** Thank you for the suggestion. Now we have made the required change the reviewer may see table 3.

13) Discussion: include citations for known barriers to accessing health facilities (lines 276-278)

**Response to reviewer:** we are thankful you for the provided comment. Now we have made the required change the reviewer may see the discussion part.

Reviewer #2: Because PLOS ONE does not copyedit accepted manuscripts; the authors should employ an editor to assist with ambiguous and grammatical errors that appear throughout the text. There are multiple grammar and sentence structure corrections that are required prior to publication.

**Response to reviewer 2:** Thank you for the comment. Now we have used professional editing services like paper true and proofreading to correct spelling, punctuation and grammatical errors.
