# Supplementary material for: Pregnant women’s intentions to use maternity waiting homes and its associated factors in rural districts of Hadiya Zone, Southern Ethiopia
Source: PLoS One. 2023 Jun 2;18(6):e0281652. doi: 10.1371/journal.pone.0281652 (PMC10237373; doi:10.1371/journal.pone.0281652)
Supplement: Attachment — Submitted filename: Response to Reviewers last.docx [file pone.0281652.s005.docx]

My coauthors and I thank you for your comments and suggestions concerning our manuscript “Pregnant women’s intentions and factors associated with maternity waiting home utilization in rural districts of Hadiya Zone, Southern Ethiopia”). We also genuinely appreciate the thoughtful comments from the reviewers, which have improved the paper.

We have studied the comments carefully and have revised our paper accordingly. This letter provides point-by-point responses to each comment and summarizes relevant changes in the manuscript. These changes are highlighted yellow in the revised manuscript. Primary changes to the paper include the following:

The reviewers have raised a number of concerns that need attention.

Could you please revise the manuscript to carefully address the concerns raised?

**Response to Editor:** Thank you for your time and consideration. We have addressed each reviewer comment and have revised the manuscript according to the reviewers’ comments.

Journal Requirements: Please review your reference list to ensure that it is complete and correct. If you have cited papers that have been retracted, please include the rationale for doing so in the manuscript text, or remove these references and replace them with relevant current references. Any changes to the reference list should be mentioned in the rebuttal letter that accompanies your revised manuscript. If you need to cite a retracted article, indicate the article’s retracted status in the References list and also include a citation and full reference for the retraction notice.

**Response:** We have reviewed and adjusted our references to ensure that they are in compliance with PLOS specifications. All changes are highlighted yellow in the revised manuscript. The editor may see the reference part.

Review Comments to the Author

Reviewer #3: Major comments;

1. Researchers used systematic sampling technique to recruit respondents. But it is not clear how the sampling frame was established? Did you do household listing? And subsequent household was contacted in cases of no pregnant woman in the selected household. It means that every pregnant woman did not have an equal chance of selection and induces bias.

**Response:** Thank you for your comment. Now we have modified it based on the given comment. The reviewer may see the method part.

2. Most previous studies identified maternal education and distance to health facility predicts use of/ intention to use MWHs. But in this study these variables are not included in the multivariable analysis. Please force retain them in the multivariable analysis and discuss and interpret possible lack of association with the outcome variable

**Response:** Thank you for the comment. We have amended it according to the given comment.
3. Number of ANC visit would possible be confounded or modified by gestational age. Please add gestation age in the analysis and possible interaction term. Otherwise, interpretation of # of ANC would be misleading and ambiguous

4. Multiple grammatical errors that need to be fixed.

**Response:** Thank you again for bringing this to our attention. We have also obtained the help of an editor to correct errors in grammar and sentence structure throughout the manuscript. Every change in the manuscript has been highlighted in yellow.

Minor comments;

1. The Title seems a qualitative study design. I would drop “Facilitators and barriers” and replace associated or predictors factors

**Response:** The title is already modified by the previous comment as (Pregnant women’s intentions and factors associated with maternity waiting home utilization in rural districts of Hadiya Zone, Southern Ethiopia). The reviewer may see the title.

2. Line 57, replace majority by most

**Response:** Thank you for the comment. Now we have made the needed change.
3. Line 58-61, update by MiniDHS 2019 findings

**Response:** Thank you for your time and consideration. We have updated the sentences in the introduction part.

4. Line 69, remove, between that and MWHs

**Response:** Thank you for the comment. Now we have corrected it according to the given comment.
5. Line 72-73, contradictory evidence regarding wealth. Check that

**Response:** Thank you for your deep insight. We understand the raised concern. But, it is correct since we have checked it.

6. Paragraph 3 is too long and difficult to catch. Reorganize/theme factors and narrate in a separate paragraph

**Response:** Thank you for the comment. Correction is made according to the raised concern. The reviewer may see the introduction part.

7. Line 92-93, significance of the study is a bit exaggerated. Discount to … would help program managers and service providers to design appropriate interventions….and provide appropriate care for pregnant women….

**Response:** Thank you for bringing this to our attention. We have updated the sentences in the introduction part.

8. Sub-title the settings, design, sample size and sampling techniques

**Response:** Thank you for the comment. Now we have made the essential correction. The reviewer may see the method part

9. Line 101, define kebele here; that means move the definition in line 105 here

**Response:** Thank you for the comment. Now we have made the necessary amendment.
10. Line 123, how fundal height is measured at home?

**Response:** Thank you for the remark. Now we have made the necessary modification.
11. Line 124-127, move it to “Measurement” section

**Response:** We are indebted for the provided comment. We have made the necessary amendment according to the given comment. The reviewer may the measurement part.

12. In the definition of terms like positive subjective norms, please give items included to measure such terms

13. Line 147, report exact value of alpha

**Response:** Thank you for the comment. We accept the suggestion and we have mentioned the exact value of alpha. The reviewer may see the method part.

14. Line 147, what are the local languages used? Hadiyigna and Amharic? Please specify

**Response:** We are grateful you for the provided comment. Now we have made the required change the reviewer may see the method part.

15. Line 149, change hadiya to Hadiya. Same comment throughout the manuscript

**Response:** Thank you very much for your comment. Now the method part is corrected as per the given suggestion. The reviewer may see the method part.

16. Line 150, specify what changes did researchers made after pre-testing

**Response:** We are thankful for the delivered statement and we have made an essential modification in this regard. The reviewer may see the method part.

17. Line 179, what type of treatment? This is confusing as this is a household survey.

**Response:** We are grateful you for the provided comment. Now we have made the required change by removing the term household survey. The reviewer may see the method part.
18. Line 183, replace majority by most

**Response:** Thank you very much for the given comment. We have corrected it according to the given comment.

19. Line 182, as researchers replace respondents/household, I don’t think there is a need to report response rate.

**Response:** Thank you very much for the given comment. Now we have modified it.

20. Line 189-190, specify obstetric complication and ANC4+ are from current or past pregnancy

**Response**: Thank you for the comment. Now we included definition for obstetric complication in measurement part besides, the necessary correction is made with regard to ANC4+.
21. Table 1, categorize marital status as in union and not in union; number of households not clear if it is to mean number of household members correct as such; time taken to reach facility in minutes; specify means of transport (it is walking, by car, etc.)

**Response:** We are indebted for the provided statement and we have made the essential modification in this regard. The reviewer may see table 1.

22. Table 2, number of ANC visits ever received; change still birth to stillbirth

**Response:** We are appreciative for the provided comment and we have made the necessary amendment in this regard.

23. Discussion section, use odds of… rather than more likely…

**Response:** Thank you for pointing this out. We have made the requested changes. The reviewer may see the discussion part.

24. Line 314-15, recall bias is a problem not only for the outcome variable but also for the predictor variables too. Please also add social desirability bias and mention techniques you applied to minimize such biases

**Response:** We are thankful for the provided comment and we have made the necessary modification on the limitation part. The reviewer may see this part.

25. Line 329, please acknowledge Hossana College of Health Science for funding the study

**Response:** We are grateful for the given comment and we have made the essential modification on the acknowledgement part. The reviewer may see this part.
