# Supplementary material for: Pregnant women’s intentions to use maternity waiting homes and its associated factors in rural districts of Hadiya Zone, Southern Ethiopia
Source: PLoS One. 2023 Jun 2;18(6):e0281652. doi: 10.1371/journal.pone.0281652 (PMC10237373; doi:10.1371/journal.pone.0281652)
Supplement: Attachment — Submitted filename: Response to Reviewers.docx [file pone.0281652.s006.docx]

**Thank you very much for your comments, suggestions and critical appraisal.**

PONE-D-21-13771R2

Pregnant women’s intentions and factors associated with maternity waiting home utilization in rural districts of Hadiya Zone, Southern Ethiopia

Thank you for submitting your manuscript to PLOS ONE. After careful consideration, we feel that it has merit but does not fully meet PLOS ONE’s publication criteria as it currently stands. Therefore, we invite you to submit a revised version of the manuscript that addresses the points raised during the review process.

Reviewers have continued to note that this manuscript could make an important contribution to the literature on maternity waiting homes. I had the pleasure of reviewing the manuscript myself during its initial evaluation. After assessing your most recent response to reviewers, I have identified multiple items raised by Reviewer 3 that have not been sufficiently addressed in your resubmission. These require a Major Revision to the manuscript. Please address the below listed items and resubmit the manuscript. I have retained Reviewer #3's original numbering below for ease of comparing with your most recent response to reviewers. I have included a few clarifying notes within parentheses.

Unaddressed Points Raised by Reviewer #3:=

1. Researchers used systematic sampling technique to recruit respondents. But it is not clear how the sampling frame was established? Did you do household listing?

**Response.** Thank you for the comment. Yes, we have performed household listing the sampling frame. Now we have made a considerable amendment. The reviewer may see the method part.

2. Most previous studies identified maternal education and distance to health facility predicts use of/ intention to use MWHs. But in this study these variables are not included in the multivariable analysis. Please force retain them in the multivariable analysis and discuss and interpret possible lack of association with the outcome variable.

**Response.** We are thankful for the given comment. Now we have interpreted possible lack of association for maternal education and distance to health facility in the discussion part. The reviewer may see the discussion portion.

3. Number of ANC visit would possible be confounded or modified by gestational age. Please add gestation age in the analysis and possible interaction term. Otherwise, interpretation of # of ANC would be misleading and ambiguous

**Response.** We are thankful for the given comment. We have added gestation age. The reviewer may see the measurement part.

9. Line 101, define kebele here; that means move the definition in line 105 here (i.e. define Kebele the first time it is mentioned in the text)

**Response.** Thank you for the comment. Correction is made according to the provided comment.

10. Line 123, how fundal height is measured at home? (i.e. gestational age)

**Response.** Thank you for the comment. We have shared the concern. Fundal height is measured since the data collectors are midwives.

12. In the definition of terms like positive subjective norms, please give items included to measure such terms (ensure items used to construct this indicator are explained within the methods).

**Response.** Thank you for the comment. At this instant, we have included the items used to measure subjective norms. You may see the measurement part.

17. Line 179, what type of treatment? This is confusing as this is a household survey. (Is this treatment at the health facility as the interviewers were midwives? This requires explanation within the text)

**Response.** We are grateful for the given comment and we have removed this word.
I concur with Reviewer #3 regarding the need to clarify gestational age within the analysis as gestational age does not appear to have been accounted for. If gestation age was an eligibility criteria it should be described in the methods as such. If it was not considered within the study, that should be explained in the limitations.

**Response.** We are thankful for the given comment. In this study, gestational age was considered. For determining gestational age we have used last menstrual period and fundal height as the data collectors are midwives.

I further concur with Reviewer #3 that "positive subjective norms" requires further explanation within the methods. While "5 items" is stated on Line 146, information on what those five items are is not provided.

**Response.** We are thankful for the given comment. Now we have included the items used to measure subjective norms. You may see the measurement part. In addition, as PLOS ONE does not offer copy-editing services at the final stage of publication, this manuscript still requires some English editing for sentence structure, grammar, and word choice. The manuscript also requires editing for minor formatting issues (e.g. double spaces within tables, incorrectly located header for Table 3, the spelling of "newborn" without a hyphen).

**Response.** Thank you for the comment. Now we have used professional editing services like paper true and proofreading to correct spelling, punctuation and grammatical errors. Formatting issues were considered according to the given comment.
