# Supplementary material for: Pregnant women’s intentions to use maternity waiting homes and its associated factors in rural districts of Hadiya Zone, Southern Ethiopia
Source: PLoS One. 2023 Jun 2;18(6):e0281652. doi: 10.1371/journal.pone.0281652 (PMC10237373; doi:10.1371/journal.pone.0281652)
Supplement: Attachment — Submitted filename: Respose to Reviewers.docx [file pone.0281652.s007.docx]

**First of all, we want to thank you very much for your detail questions, comments and suggestions.**

**PONE-D-21-13771R3**

**Pregnant women’s intentions and factors associated with maternity waiting home utilization in rural districts of Hadiya Zone, Southern Ethiopia**

**Additional Editor Comments:**

Thank you for your submitted revisions. Comments from the reviewers are included below. The reviewers advise additional copy editing of the manuscript for grammar, spelling, and spacing. As PLOS ONE does not provide copy editing services before publication, this is the responsibility of the authors. Please ensure all grammatical, spelling, and formatting errors are corrected with resubmission of your manuscript.

**Response to editor:** We are very grateful for the comment you provided. Now, as much as possible, we have tried to correct language usage flaws including punctuations, wordings, spelling and grammar errors using English editing service.

**Reviewers' comments:**

**Reviewer #2:** Grammatical, spelling and spacing errors throughout the manuscript.

**Response to reviewer:** We are grateful for the suggestion. At this instant we have used English editing service to correct spelling, punctuation and grammatical errors. The reviewer may see the yellow highlighted part mainly on the introduction and discussion part.

In response to reviewer #3, the authors have added definitions of terms but they seem misplaced in the manuscript. I would recommend using a secondary section in the methods section where these definitions are listed and not just dropped into the methods section.

**Response to reviewer:** We are very thankful for the comment you delivered. In this instant we have written in a secondary section of method part. The reviewer may see the method part on page 7 and 8.

Please add information on the informed consent process of participants within the data collection description for better flow.

**Response to reviewer:** Thank you for the provided comment. By understanding your concern, we have modified the informed consent process to make it coherent. The reviewer may see the method part on page 9.

How is “wealth status” calculated/defined?

**Response to reviewer:** The household wealth variable was created using principal components analysis of items listed in below. Items were selected to minimize clustering and truncation which compromise reliability An asset-based wealth index created using information on asset ownership (radio, television, mobile phone, motorbike, car/truck), number of animals owned (cows, sheep, poultry), electricity supply to home, health insurance, drinking water source, type of toilet and type materials used for construction of floors in the home. It was divided into three groups: low (poor), medium, and high (rich) [26].

26. Vyas S, Kumaranayake L. Constructing socio-economic status indices: how to use principal components analysis. Health Policy Plan 2006; 21:459-68. The reviewer may see the method part on page 9.

Line 297-299: “This finding implies that, as long as the issue of service quality is addressed, women in the wealthier quintiles can be encouraged to use MWHs.” I don’t believe the authors provide the data to support this statement.

**Response to reviewer:** Thank you for the given comment. We have understood the raised concern. Now we have corrected agreeing to the provided comment. According to the findings of the current study, poor pregnant women have 2.52 times higher probabilities of intending to use MWHs than their counterparts. This is in line with a study carried out in Tanzania [11]. The reason could be related to the fact that for poorer women, the MWHs may be a means to access higher level obstetric care without incurring costs for private transport during labor and delivery. However, this result is in contrast with the studies conducted in Northwest Ethiopia, Jimma zone, and Timor Leste, respectively [8, 9, 23]. The reason could be the wealthiest women have the economic means for emergency transport to the hospital once labor starts. The reviewer may see the discussion part.

8. Endayehu M, Yitayal M, Debie A. Intentions to use maternity waiting homes and associated factors in Northwest Ethiopia. BMC Pregnancy Childbirth.2020;20(1):1-10. https://doi.org/10.1186/s12884-020-02982-0.

9. Kurji J, Gebretsadik LA, Wordofa MA, et al Factors associated with maternity waiting home use among women in Jimma Zone, Ethiopia: a multilevel cross-sectional analysis BMJ Open 2019;9:e028210. https://bmjopen.bmj.com/content/9/8/e028210

23. Wild K, Barclay L, Kelly P, Martins N. The tyranny of distance: maternity waiting homes and access to birthing facilities in rural Timor-Leste. Bull World Health Organ. 2012; 90:97- 103. https://doi.org/10.2471/BLT.11.088955. PMID: 22423160

**Reviewer #3: Minor comments**

1. This paper would benefit from an academic editor

**Response to reviewer:** We are very grateful for the comment you provided. Now, as much as possible, we have tried to correct language usage flaws including punctuations, wordings, spelling and grammar errors using English editing service. The reviewer may see the yellow highlighted part mainly on the introduction and discussion part.

2. Fix grammatical errors such as line 84, change the word moms to mothers or women;

**Response to reviewer:** Now we have replaced the word mothers by moms. The reviewer may see the introduction part on page 5.

Line 85, don't hyphenate newborns;

**Response to reviewer:** Now we have removed the hyphen. The reviewer may see the introduction part which is highlighted in yellow color.

Line 92, remove double ","; .

**Response to reviewer:** Now we have removed the hyphen. The reviewer may see the introduction part which is highlighted in yellow color.

**Response to reviewer:** Now we have removed the unnecessary commas. The reviewer may see the introduction part.

Line 116, change the word are by were and specify the period;

**Response to reviewer:** We have made correction according to the comment. The reviewer may see the method part on page 6.

Line 120 change the word Health Centers to health centers;

**Response to reviewer:** We have made a correction by avoiding unnecessary capitalization. The reviewer may see page 6.

Line 133-134, the sentence is not clear (is that to mean proportional allocation was made to Kebeles?);

**Response to reviewer:** Proportional allocation was made to the number of households. The k-value of four was obtained by dividing the source population (1540) by the sample size (385). The reviewer may see page 6 on the method part for more clarification.

Line 149 drop "for institutional";

**Response to reviewer:** We have accepted the comment and dropped in to new line. The reviewer may see page 6.

Line 169 and 173 change the word was to were;

**Response to reviewer:** We have made a correction**.** The reviewer may see page 8.

Line 219 change multifarious to multiparous;

**Response to reviewer:** We have replace the word multiparous by multifarious. The reviewer may see page 10.

Line 309 change the word above to more.

**Response to reviewer:** we have replaced the word above by more. The reviewer may see page 6

3. Line 129, the source population is 1.65 million; why the authors applied finite population correction is not clear.

**Response to reviewer:** As stated by the reviewer, 1.65 million is not a source population. It is an overall population found in Hadiya zone. Instead, a total of 1540 pregnant women were reported as a source population from selected kebeles. That’s why we have used a finite population correction formula. The reviewer may see page 6 on method part.

4. Line 134-137, the sampling interval is not specified; still it is not clear how researchers locate households once systematically selected. Did researchers use local guiders or HEWs to locate households? Please specify

**Response to reviewer:** Proportional allocation was made to the number of households. The sampling interval (k-value) of four was obtained by dividing the source population (1540) by the sample size (385). We have used HEWs to locate households. The reviewer may see the method part on page 6.

5. Drop some of the operational definitions like SFH; nothing adds to this paper.

**Response to reviewer:** We have added the SFH as it was asked by other reviewer during the previous revisions that is why we have incorporated it.

6. Line 225, indicate these percentages are from those who intend to stay at MWHs, not from the whole sample

**Response to reviewer:** Thank you for your deep insight. We understand the raised concern. Now we have calculated the percentage of favorable attitude from women who had an intention to use MWH not from overall intention to use MWH). We have made the necessary modification according to the given comment. The reviewer may see page 10 on the result part and table 3.

7. Table 3, the values for the variable "distance to health facility" is not correct; please fix it; also add percentage values

**Response to reviewer:** We have checked the value of the variable mention which is correct. But, we forgot to incorporate its percentage. Now we included its percentage. The reviewer may see table 3.
