# Supplementary material for: Pregnant women’s intentions to use maternity waiting homes and its associated factors in rural districts of Hadiya Zone, Southern Ethiopia
Source: PLoS One. 2023 Jun 2;18(6):e0281652. doi: 10.1371/journal.pone.0281652 (PMC10237373; doi:10.1371/journal.pone.0281652)
Supplement: Attachment — Submitted filename: Respose to Reviewers.docx [file pone.0281652.s008.docx]

**Pregnant women’s intentions to use maternity waiting homes and its associated factors in rural districts of Hadiya Zone, Southern Ethiopia**

First of all, we want to thank you both the editor and reviewers for your detail questions, comments and suggestions. We strongly believe without your significant and valuable expertise the manuscript will not be in this stand.

**PONE-D-21-13771R4**

**Additional Editor Comments:**

Please review your reference list to ensure that it is complete and correct. If you have cited papers that have been retracted, please include the rationale for doing so in the manuscript text, or remove these references and replace them with relevant current references. Any changes to the reference list should be mentioned in the rebuttal letter that accompanies your revised manuscript. If you need to cite a retracted article, indicate the article’s retracted status in the References list and also include a citation and full reference for the retraction notice.

**Response to editor:** Fist of all, we are grateful for your valuable comments and suggestions. We have checked and reviewed all the references list and we haven’t found problem with it.

**Reviewers' comments:**

This paper would benefit from an academic editor

**Response to reviewer 3.** Always we wonder on the critical insight of the reviewer. Thank very much for all your effort and commitment. Now, as much as possible, we have tried to correct language usage flaws including punctuations, wordings, spelling and grammar errors using English editing service.

Title: change to "Pregnant women’s intentions to use maternity waiting homes and its associated factors in rural districts of Hadiya Zone, Southern Ethiopia"

**Response to reviewer 3.** We have agreed up the suggested modification on the title. The reviewer may see the title highlighted in yellow color.

• Still, this needs to be edited by an academic editor. Please also fully address the comments. For instance, the following grammatical errors are not addressed:

**Response to reviewer 3.** We have tried to improve language usage flaws including punctuations, wordings, spelling and grammar errors using English editing service.

Line 84, change the word “moms” to “mothers or women”;

**Response to reviewer 3.** We have replaced the word moms by mothers. The reviewer may see line- in the introduction part which is highlighted in yellow color.

Line 85, don't hyphenate newborns;

Response to reviewer 3. By accepting the raised concern we have removed the hyphen. The reviewer may see the introduction part which is highlighted in yellow color.

Line 116, change the word “are” to “were” and specify the period;

**Response to reviewer 3**. The given remark is acknowledged and amendment has been made convening to the given recommendation. The reviewer may see the method part which is highlighted in yellow color.

Line 149 drop "for institutional";

**Response to reviewer 3.** The advised comment has been accepted and modified accordingly. The reviewer may see the method part highlighted in yellow color.

Line 170 change the word “was” to “were”;

**Response to reviewer 3.** The recommended comment has been accepted and modified accordingly by replacing the word was by were. The reviewer may see the method part.

• Line 26, change the word "raise" to "expand";

**Response to reviewer 3.** Now we have replaced the word moms by mothers. The reviewer may see the abstract part highlighted in yellow color.

Line 28, drop the phrase "across the world"'

The proposed comment has been accepted and modified accordingly. The reviewer may see the abstract part highlighted in yellow color.

Line 69, drop the word "dangerously";

**Response to reviewer 3.** The suggested comment has been accepted and now totally we have removed it because its presence is not as such important. The reviewer may see the introduction part on page which is highlighted in yellow color.

Line 75-78, change by "Furthermore, only about half women were delivered by a skilled provider and 34% of women received a postnatal care check-up within the first two days after birth [3].”

**Response to reviewer 3.** The suggested comment has been accepted and modified accordingly. The reviewer may see the introduction part which is highlighted in yellow color.

Line 76, change "Antenatal" to "antenatal";

**Response to reviewer 3.** The recommended comment has been accepted and modified accordingly by replacing the word was by were. The reviewer may see the method part.

Line 166, drop the word "LMP";

**Response to reviewer 3.** We have accepted the comment and dropped in to new line. The reviewer may see the method part which is highlighted in yellow color.

Lines 92-93, drop "giving birth in a health facility, receiving skilled care from a trained provider [17, 19]" No temporal relationship between use of MWHs and skilled delivery, unless we asked for previous skilled assistance.

**Response to reviewer 3.** We have accepted the comment and dropped in to new line. The reviewer may see the introduction part which is highlighted in yellow color

Table 3, the percentage values for education, distance to health facility, and attitude could not add up to 100%; please fix it by adding column percentages similar to other variables

**Response to reviewer 3.** We have agreed up on the raised concern and made a necessary modification regarding percentage of the mentioned variables. The reviewer may see table 3 which is highlighted in yellow color.

Line 132-33, the sentence “Proportional allocation was made to the number of households.” is not clear and how many kebeles were included in the study is not clear. Please replace this with the following sentences in your original manuscript “A lottery method was used to pick ten rural kebeles. According to the approximate number of pregnant women in each kebele, the sample size was distributed proportionally.”

**Response to reviewer 3.**

• Check again or consult a statistician about the finite population correction. I think it is applied when the target population (i.e., the entire pregnant women for which the survey data are to be used to make inferences) not the source population (i.e., pregnant women residing in the PSU) is finite

**Response to reviewer 3.** We have consulted a statistician regarding population correction formula. But, we have not received a different idea with the statistician in this regard.
